# Asparagus Polysaccharide inhibits the Hypoxia-induced migration, invasion and angiogenesis of Hepatocellular Carcinoma Cells partly through regulating HIF1α/VEGF expression via MAPK and PI3K signaling pathway

**DOI:** 10.7150/jca.51407

**Published:** 2021-05-10

**Authors:** Wei Cheng, Ziwei Cheng, Lingling Weng, Dongwei Xing, Minguang Zhang

**Affiliations:** 1 Shanghai Municipal Hospital of Traditional Chinese Medicine, Shanghai University of Traditional Chinese Medicine, Shanghai 200071, China.; 2 Nanjing Hospital of Chinese Medicine affiliated to Nanjing University of Chinese Medicine, Nanjing 210001, China.

**Keywords:** HIF-1α, VEGF, asparagus polysaccharide, hepatocellular carcinoma

## Abstract

**Aim:** Although there are so many treatment strategies used for hepatocellular carcinoma (HCC), the overall survival (OS) of HCC patients still remains very low. In our previous studies, asparagus polysaccharide (ASP) has been demonstrated to suppress proliferation, migration, invasion and angiogenesis of HCC cells under normoxic conditions *in vitro*. However, the inhibitory effects of ASP on the hypoxia-induced migration, invasion and angiogenesis of HCC cells still remain largely unexplored.

**Materials and methods:** Cell Counting Kit-8 (CCK-8) assay, transwell assay, and tube formation assay were used to determine the effects of ASP on hypoxia-induced proliferation, migration, invasion and angiogenesis of HCC cells. ELISA, Western blotting analysis and immunofluorescence assay were used to confirm the effects of ASP on the expressions of HIF-1α and VEGF at the protein level. Moreover, effects of ASP on signaling pathway-related proteins were investigated by Western blotting analysis. Immunohistochemistry (IHC) assay was applied to test the effects of ASP on angiogenesis-associated proteins of tumor cells.

**Results:** We showed that ASP effectively suppressed hypoxia-induced proliferation, migration, invasion and angiogenesis of SK-Hep1 and Hep-3B cells in a dose-dependent manner. In addition, the inhibitory effect of ASP might be partly attributed to down-regulation of HIF1α and VEGF proteins in SK-Hep1 and Hep-3B cells under hypoxic conditions. Moreover, signaling pathway study indicated that ASP significantly down-regulated the hypoxia-induced expressions of p-AKT, p-mTOR and p-ERK, while it had little effects on AKT, mTOR and ERK. Besides, SK-Hep1 xenograft tumor models in nude mice further confirmed that the inhibitory effect of ASP on xenograft tumors might be exerted partly via down-regulation of HIF1α and VEGF through blocking MAPK and PI3K signaling pathways.

**Conclusions:** Our findings suggested that ASP suppressed the hypoxia-induced migration, invasion and angiogenesis of HCC cells partly through regulating HIF-1α/VEGF expression via MAPK and PI3K signaling pathways.

## Introduction

Hepatocellular carcinoma (HCC) has been ranked as the sixth common tumor and the fourth leading cause of cancer-related death worldwide 1. Although so many treatment strategies, including liver tumor resection, liver transplantation, radiofrequency ablation, transcatheter arterial chemoembolization (TACE), radiotherapy, chemotherapy, molecular targeted therapy and immune therapy, have been used for HCC patients, their overall survival (OS) still remains very low 2. The possible reason of low OS for HCC patients can be attributed to overexpression of hypoxia-inducible factor 1 (HIF-1) in liver cancer tissue 3.

HIF-1 is found by Semenza when performing human erythropoietin gene expression in 1992 4. HIF-1 is a dimeric protein containing an anoxic-sensitive alpha subunit and a persistently expressed beta subunit, of which HIF-1α is the only oxygen regulatory subunit that determines the HIF-1 activity 5, 6. HIF-1α is considered to be a central promoter of tumor hypoxia adaptive response. Under normoxic conditions, HIF-1α is hydroxylated by prolyl hydroxylase (PHD), and then binds to von Hippel-Lindau, which is then recognized by E3 ubiquitin ligase to cause its degradation 7. Under hypoxic conditions, more HIF1α is produced due to inactivation of PHD and activation of MAPK and PI3K signaling pathways 8-10. A large number of HIF-1α enter the nucleus, bind to HIF-1β, activate downstream hypoxia-response elements, then up-regulate the expressions of vascular endothelial growth factor (VEGF), angiogenin and epidermal growth factor at the protein level, and finally promote tumor cell proliferation, migration, invasion and angiogenesis 11, 12. HCC is a kind of highly vascularized tumor. Recently, many researches have demonstrated that HIF-1α could activate transcription of some genes involved in angiogenesis, glucose metabolism, proliferation, invasion, metastasis and immune escape in HCC 13.

As a very common traditional Chinese medicine (TCM), asparagus is often used to treat malignant tumors, such as breast cancer and lung cancer, by traditional Chinese oncologists. Asparagus polysaccharide (ASP) is an effective ingredient extracted from asparagus. A study has shown that ASP can enhance the phagocytosis of mouse peritoneal macrophages and increase the weight of immune organs, such as thymus and spleen 14. Another study has shown that ASP can trigger anti-tumor immune response by inducing apoptosis of myeloid-derived suppressor cells via toll-like receptor 4 15.

Our previous studies have demonstrated that ASP can induce apoptosis of HCC cells, and embolize rat hepatic artery to cause ischemic necrosis of tumor 16-18. Our recent study has indicated that ASP can suppress the migration, invasion and angiogenesis of HCC cells partly by down-regulating the HIF-1α/VEGF signaling pathway under normoxic conditions *in vitro* 19-20. However, no studies have investigated the effects of ASP on hypoxia-induced migration, invasion and angiogenesis of HCC cells (SK-Hep1 and Hep-3B cells). In order to better clarify the possible mechanism underlying the inhibitory effects of ASP on hypoxia-induced HCC cells, we performed this study in SK-Hep1 and Hep-3B cells.

## Methods

### Materials and reagents

Dulbecco's modified Eagle's medium (DMEM) and penicillin/streptomycin were obtained from Hyclone (Logan, USA). Foetal bovine serum (FBS) was purchased from Gibico (South America). Antibodies against HIF-1α and CD34 were supplied by Abcam (Cambridge, United Kingdom). VEGF antibody was provided by R&D (Minneapolis, USA). AKT, p-AKT, mTOR, p-mTOR, ERK and p-ERK antibodies were obtained from CST (Boston, USA). Horseradish peroxidase (HRP)-conjugated secondary antibodies, fluorescent-conjugated secondary antibodies, new super ECL assay, and β-actin antibody were purchased from KeyGEN BioTECH (Nanjing, China). Transwell chambers and matrigel were supplied by Corning Life Sciences (8-μm pores, Tewksbury, MA, USA) and BD Biosciences (10.5 mg/ml, San Jose, CA, USA), respectively. The Cell Counting Kit-8 (CCK-8) was obtained from Dojindo Molecular Technologies, Inc. (Kumamoto, Japan). The enzyme-linked immune sorbent assay (ELISA) kit for human VEGF was purchased from Multi Sciences (Shanghai, China). Bicinchoninic acid (BCA) protein assay kit was supplied by Beyotime Biotechnology. BALB/c nude mice were provided by Shanghai Jiesijie Experimental Animal Co., Ltd. (Shanghai, China). All animal experiments involved in our study were performed according to guidelines of institutional animal care and use committee (IACUS) and approved by the Ethics Committees of Shanghai Municipal Hospital of Traditional Chinese Medicine, Shanghai University of Traditional Chinese Medicine.

### Cell culture

Human umbilical vein endothelial cells (HUVECs), HCC cells (SK-Hep1 and Hep-3B), human normal liver cells (L0-2), and human lung adenocarcinoma cells (A549) were obtained from the Type Culture Collection of the Chinese Academy of Sciences (Shanghai, China), and the cells were maintained in DMEM supplemented with 10% FBS and 1% penicillin-streptomycin at 37 °C in a humidified atmosphere containing 5% CO_2_. In addition, cell culture conditions of 5% CO_2_, 94% N_2_ and 1% O_2_ were defined as the hypoxic culture. The medium was changed for three times every week.

### Preparation of ASP

ASP was obtained from Shanghai Yuanye Bio-Technology Co, Ltd (Shanghai, China) and dissolved in DMEM to a final concentration of 200 mg/ml. Subsequently, the supernatant was obtained by centrifugation at 200×g for 5 min and then stored at -20 °C for long-term use.

### Cell viability assay

Briefly, SK-Hep1, Hep-3B, L0-2, and A549 cells were seeded into 96-well plates at a density of 1×10^4^ cells/well and treated with different concentrations of ASP under hypoxic conditions for 24 h. Cell viability was determined by CCK-8 using a microplate reader. The relative inhibition rate of cell viability was calculated according to the formula R = [(A2-A1)/A2] ×100%, where R is the relative inhibition rate, A1 is the mean absorbance value of cells treated with ASP, and A2 is the mean absorbance value of cells in the absence of drug.

### Transwell migration and invasion assays

The bottom chambers of the transwell apparatus were filled with 600 µL DMEM supplemented with 20% FBS. The top chambers of transwell apparatus were coated with 100 µL diluted matrigel (1: 3) for 30 min in a cell incubator for subsequent invasion assay, and then 5×10^4^ HCC cells (SK-Hep1 and Hep-3B) pre-treated with various concentrations of ASP (0, 2.5, 5 and 10 mg/ml under hypoxic conditions, and 0 mg/ml under normoxic conditions) for 24 h were loaded. The plates used for migration assay and invasion assay were incubated at 37 °C for 24 h and 72 h, respectively. Images were acquired using inverted microscope, and migrated and invasive cells were quantified by manual counting.

### Tube formation assay of HUVECs

HCC cells (SK-Hep1 and Hep-3B) were exposed to different concentrations of ASP (0, 2.5, 5 and 10 mg/ml under hypoxic conditions, and 0 mg/ml under normoxic conditions) for 24 h. Subsequently, the cell supernatants were collected as conditioned medium for subsequent analysis. Each well of the 96-well plates was coated with 50 µL matrigel and incubated at 37 °C for 30 min, and then 100 µL conditioned medium was dispensed. HUVECs (2.5×10^4^ cells) were seeded on the matrigel and incubated at 37 °C. After 24 h of incubation, the tubular structure formation of endothelial cells was photographed, and the tube number was quantified using Image J.

### ELISA

HCC cells (SK-Hep1 and Hep-3B) were exposed to various concentrations of ASP (0, 2.5, 5 and 10 mg/ml under hypoxic conditions and 0 mg/ml under normoxic conditions) for 24 h. Subsequently, ELISA kit was used to detect the level of secreted VEGF in the culture supernatant of HCC cells according to the manufacturer's instructions.

### Western blotting analysis

SK-Hep1 and Hep-3B cells were pre-treated with different concentrations of ASP (0, 2.5, 5 and 10 mg/ml under hypoxic conditions, and 0 mg/ml under normoxic conditions) for 24 h. The whole cells were lysed with RIPA lysis buffer containing protease inhibitors and phenylmethylsulfonyl fluoride for 30 min on ice. The protein content was determined using a BCA protein assay kit. Equal amounts of proteins (50-100 μg) were subjected to SDS-PAGE and then electrotransferred onto a nitrocellulose membrane. Subsequently, the membranes were blocked with 5% non-fat milk at room temperature for 2 h and then incubated with respective primary antibodies (1:1,000) overnight at 4 °C, followed by incubation with HRP-conjugated secondary antibodies (1:2,000) at room temperature for 1 h. Next, immunoreactive bands were visualised using a new super ECL assay and then exposed to film.

### Immunofluorescence assay

HCC cells pre-treated with various concentrations of ASP (0, 2.5, 5 and 10 mg/ml under hypoxic conditions, and 0 mg/ml under normoxic conditions) were seeded onto glass coverslips, and then fixed in 4% paraformaldehyde (PFA) for 15 min at room temperature. The samples were blocked with blocking buffer containing 3% FBS, 1% goat serum and 0.1% Triton X-100 at room temperature for 2h. Then, cells were incubated overnight at 4 °C with the primary antibodies (1: 200), followed by incubation with fluorescent-conjugated secondary antibodies (1:1,000) at room temperature for 1 h. Subsequently, cells were incubated with DAPI at room temperature for 10 min. Finally, the fluorescence was detected with a fluorescence microscope.

### Xenograft model and drug treatment

A total of 20 male BALB/c nude mice (4-6 weeks old, Shanghai Jiesijie Experimental Animal Co., Ltd., China) were used in this study and randomly divided into four groups as follows: high-concentration ASP group (HASP, 100 mg/kg/day), medium-concentration ASP group (MASP, 50 mg/kg/day), low-concentration ASP group (LASP, 25 mg/kg/day) and PBS group (PBS, 0 mg/kg/day). Furthermore, tumors were established via subcutaneous injection of Sk-Hep1 cells (5×10^6^ cells per animal) into the right flank of the nude mice, which were administered with different concentrations of ASP by intragastric injection after 12 days. The tumor volume of the xenograft tumors and the body weight of nude mice were recorded every 3 days for 5 weeks and calculated according to the formula V = 0.5×L×W^2^ (V, volume; L, length; W, width). All animals were sacrificed by euthanasia, and the xenograft tumors were obtained for the immunohistochemistry (IHC) assay and western blotting analysis.

All animal experiments involved in our study were carried out according to institutional guidelines and approved by the Ethics Committees of Shanghai Municipal Hospital of Traditional Chinese Medicine, Shanghai University of Traditional Chinese Medicine.

### IHC staining

Paraffin-embedded sections were deparaffinised in an oven, hydrated and then treated with 3% H_2_O_2_ to block endogenous peroxidase activity, followed by thermal repair. After cooling, the sections were blocked with 10% normal goat serum for 1 h at room temperature. Subsequently, the sections were incubated with antibodies against HIF-1α, VEGF and CD34 (diluted at 1: 200) overnight at 4 °C, followed by incubation with HRP-conjugated secondary antibodies (1:600) at room temperature for 1 h. Finally, all sections were counterstained with diaminobenzidine and Mayer's haematoxylin.

### Statistical analysis

All data were presented as the mean ± standard deviation, and the difference between groups was analyzed by two-tailed analysis of variance (ANOVA). Repeated measures ANOVA was used to compare the volume of tumor and the body weight of nude mice among all groups. Statistical analyses were performed by using SPSS Statistics 21, and *P*<0.05 was considered as statistically significant.

## Results

### ASP suppresses the proliferation, migration and invasion of HCC cells and the tube formation of HUVECs under hypoxic conditions

**Figure 1:**
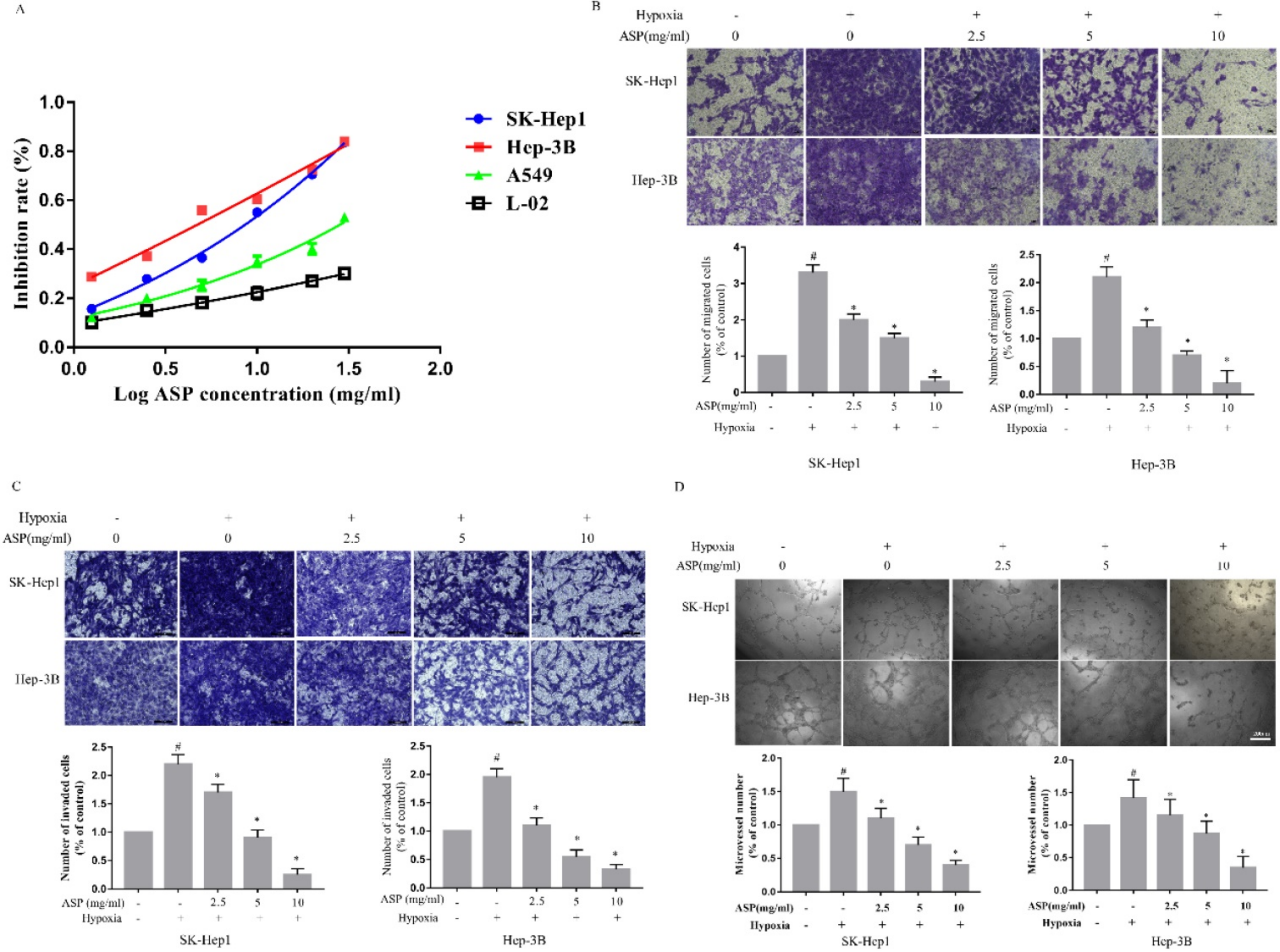
Effects of ASP on the proliferation, migration and invasion of HCC cells and the tube formation of HUVECs under hypoxic conditions. **(A)** CCK-8 assay was performed to test the effect of ASP (30, 20, 10, 5, 2.5, 1.25, and 0 mg/ml under hypoxic conditions, for 24h) on proliferation in SK-Hep1, Hep-3B, L0-2 and A549 cells.** (B-C)** Transwell migration and invasion assay were performed to test the effect of ASP (0, 2.5, 5 and 10 mg/ml under hypoxic conditions and 0 mg/ml under normoxic conditions) on migration for 24 h and on invasion for 72h in SK-Hep1 and Hep-3B cells. Cells on the lower surface of the transwell membrane were stained with 0.1% crystal violet; Histogram presented the average number of migrated and invasive cells per field; Scale bar, 50 µm in Figure B and 100 µm in Figure C.** (D)** The medium from SK-Hep1 and Hep-3B cells pre-treated with ASP (0, 2.5, 5, and 10 mg/ml under hypoxic conditions and 0 mg/ml under normoxic conditions, for 24 h) was collected and then applied to tube formation assay; Representative histogram of the area covered by the tube network quantitated using Image-Pro Plus software; Scale bar, 200 µm in Figure D. #,* p* < 0.05, compared with the normoxic control group. *, *p* < 0.05, compared with the hypoxic control group. Data were graphed using GraphPad Prism 5.02.

CCK-8 was used to detect the inhibitory effects of different concentrations of ASP on the proliferation of SK-Hep1, Hep-3B, L0-2, and A549 under hypoxic conditions for 24 h. We showed that ASP could significantly suppress the proliferation of HCC cells in a dose-dependent manner (Figure 1A). Additionally, ASP could weakly inhibit proliferation of A549 and only a little effects on L0-2 (Figure 1A). The IC_50_ values of ASP were 4.76 mg/ml and 8.18 mg/ml for SK-Hep1 and Hep-3B cells, respectively. Therefore, 10 mg/ml, 5 mg/ml and 2.5 mg/ml were chose as high, medium and low concentrations for the subsequent experiments, respectively. Migration and invasion play an important role in process of tumor progression. Inhibition of migration and invasion for tumor cells may block the tumor metastasis to a certain extent. As shown in Figure 1B-C, the ability of migration and invasion of HCC cells (SK-Hep1 and Hep-3B) were significantly enhanced after hypoxic challenge compared with the normoxic conditions, and ASP could effectively suppress the hypoxia-induced migration and invasion of HCC cells in a dose-dependent manner. Angiogenesis was considered as a very complex process, in which tube formation of HUVECs was an important step. Our results demonstrated that the ability of HCC cells (SK-Hep1 and Hep-3B)-induced tube formation of HUVECs was significantly enhanced after hypoxic challenge compared with the normoxic conditions, while ASP could effectively suppress the HCC-induced tube formation of HUVECs under hypoxic conditions in a dose-dependent manner (Figure 1D).

### ASP suppresses the expressions of HIF-1α and VEGF at the protein level in HCC cells under hypoxic conditions

**Figure 2:**
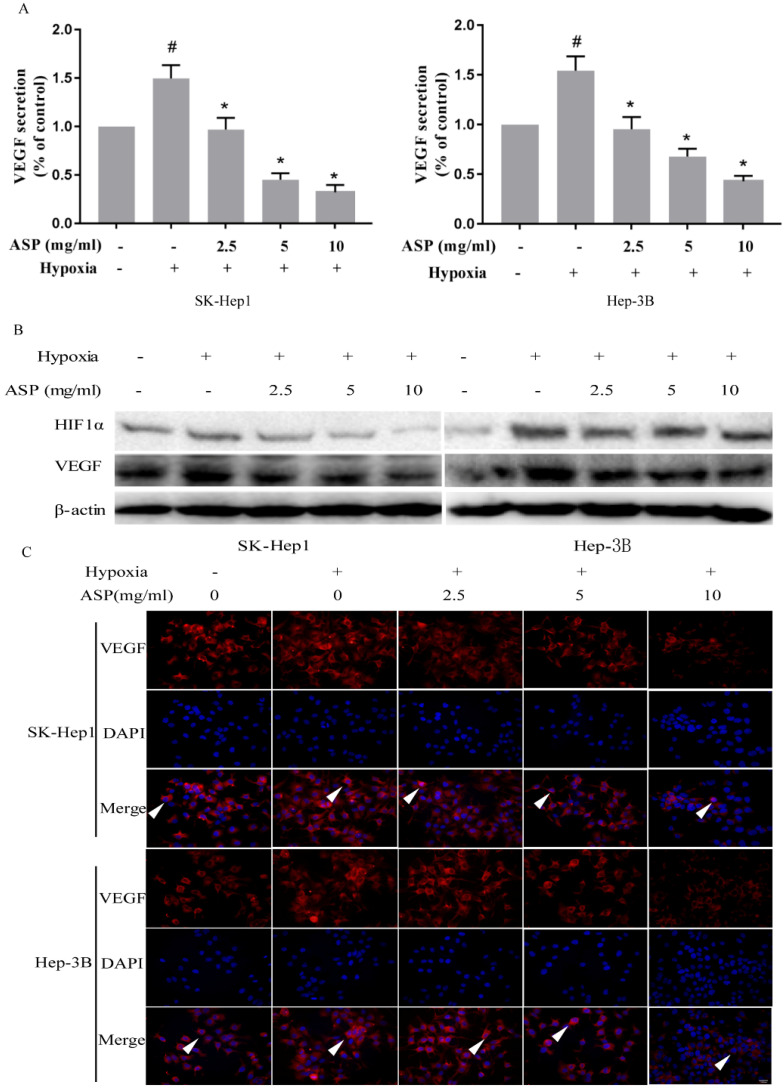
Effects of ASP (0, 2.5, 5, and 10 mg/ml under hypoxic conditions, and 0 mg/ml under normoxic conditions, for 24 h) on the expressions of HIF-1α and VEGF in SK-Hep1 and Hep-3B cells. **(A)** Secreted VEGF protein was analyzed by ELISA; **(B)** Protein expressions of HIF-1α and VEGF were analyzed by Western blotting analysis, and β-actin was used as a loading control; **(C)** Protein expression of VEGF was analyzed by immunofluorescence assay; Scale bar, 100 µm in Figure C. The white arrows signify merged fluorescence cells. #,* p* < 0.05, compared with the normoxic control group. *, *p* < 0.05, compared with the hypoxic control group.

In order to explore inhibitory mechanism of ASP on migration, invasion and angiogenesis of HCC cells under hypoxic conditions, we assessed the expressions of HIF-1α and VEGF at the protein level in HCC cells under hypoxic conditions using ELISA, Western blotting analysis, and immunofluorescence assay. We used ELISA kit to detect the VEGF secretion in the culture supernatant of HCC cells (SK-Hep1 and Hep-3B) and found that the VEGF secretion of HCC cells was significantly increased upon hypoxic challenge compared with the normoxic conditions, while ASP could effectively suppress the VEGF secretion of HCC cells under hypoxic conditions in a dose-dependent manner (Figure 2A). Moreover, Western blotting analysis was used to investigated the effects of ASP on the expressions of HIF-1α and VEGF at the protein level in HCC cells (SK-Hep1 and Hep-3B) under hypoxic conditions, and we found that the expressions of HIF-1α and VEGF at the protein level were significantly up-regulated in HCC cells upon hypoxic challenge compared with the normoxic conditions, while ASP could effectively suppress such up-regulation of HIF-1α and VEGF in a dose-dependent manner (Figure 2B). Additionally, we used immunofluorescence assay to detect the effects of ASP on the VEGF expression in HCC cells (SK-Hep1 and Hep-3B) under hypoxic conditions, and we found that the expression of VEGF at the protein level was significantly up-regulated in HCC cells upon hypoxic challenge compared with the normoxic conditions, while ASP could effectively suppress the expression of VEGF at the protein level in HCC cells under hypoxic conditions in a dose-dependent manner (Figure 2C).

### Inhibitory effects of ASP on the expressions of HIF-1α and VEGF under hypoxic conditions may be partly mediated by down-regulating the phosphorylation of MAPK and PI3K signaling pathway-related proteins in HCC cells

**Figure 3:**
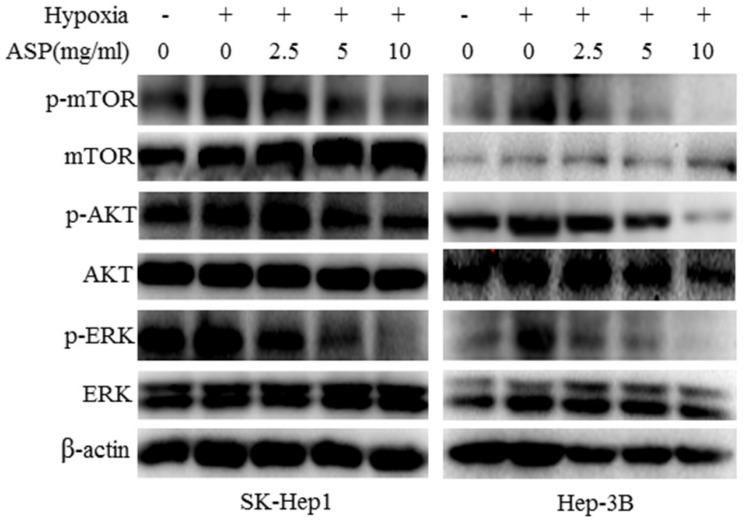
Effects of ASP (0, 2.5, 5 and 10 mg/ml under hypoxic conditions and 0 mg/ml under normoxic conditions for 24 h) on the protein levels of AKT, p-AKT, mTOR, p-mTOR, ERK and p-ERK in SK-Hep1 and Hep-3B cells were analyzed by Western blotting analysis, and β-actin was used as a loading control.

It is well known that MAPK and PI3K signaling pathways play an important role in regulating the expressions of HIF-1α and VEGF at the protein level in cancer cells 20-22. Our previous results have shown that ASP can significantly inhibit MAPK and PI3K signaling pathways in HCC cells under normoxic conditions 20. In order to further determine the effects of ASP on MAPK and PI3K signaling pathways in HCC cells (SK-Hep1 and Hep-3B) under hypoxic conditions, we examined the protein levels of AKT, p-AKT, mTOR, p-mTOR, ERK and p-ERK after ASP treatment for 24 h under hypoxic conditions in SK-Hep1 and Hep-3B cells. Our data indicated that the protein levels of p-AKT, p-mTOR and p-ERK were significantly up-regulated upon hypoxic challenge compared with the normoxic conditions, while ASP significantly suppressed those protein expressions under hypoxic conditions (Figure 3). There were no significant differences in the protein levels of AKT, mTOR and ERK under hypoxic conditions after ASP treatment (Figure 3).

### ASP suppresses the growth and angiogenesis of subcutaneous xenograft tumors in nude mice

**Figure 4:**
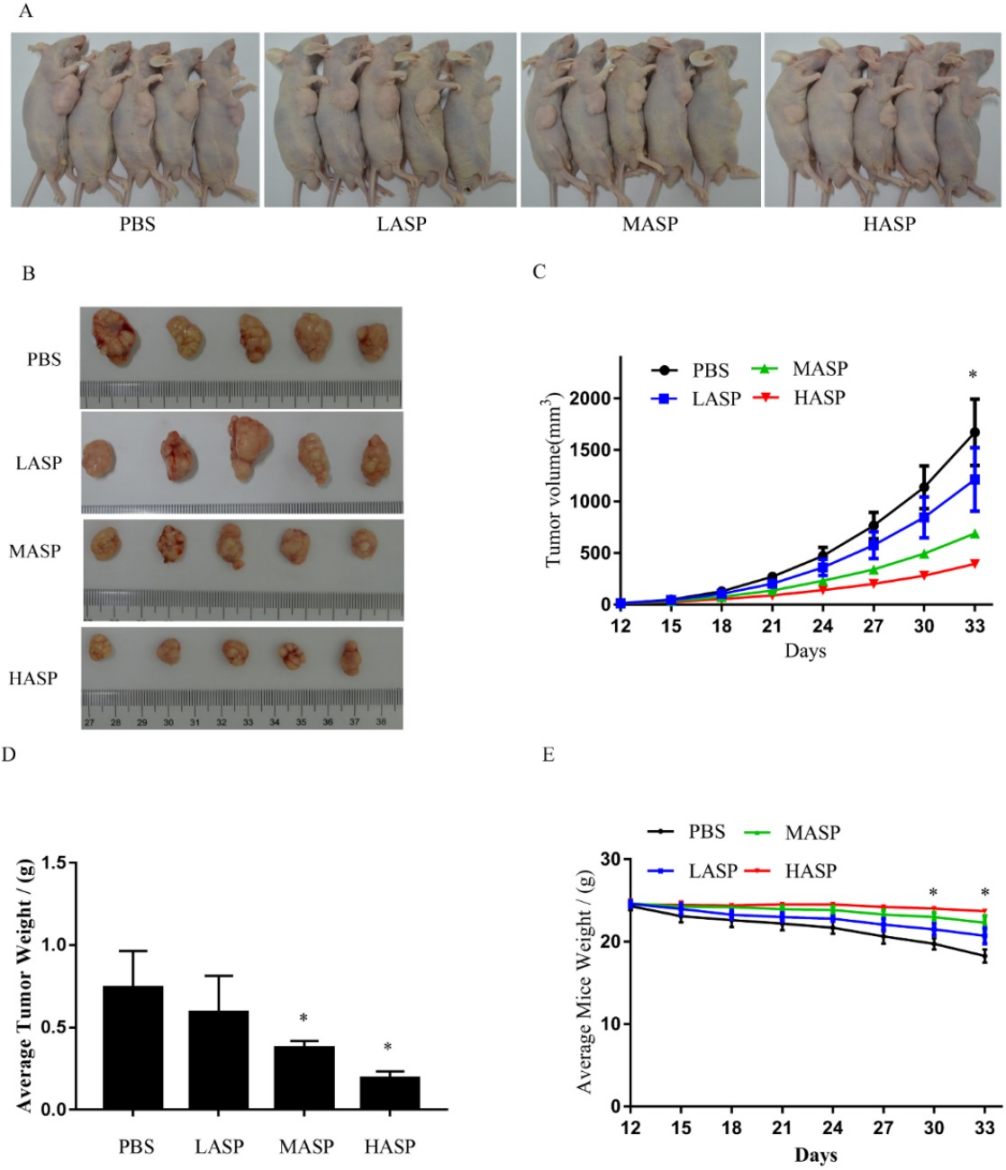
ASP suppresses the growth of subcutaneous xenograft tumors and prevents body weight loss in nude mice. **(A)** Representative images of nude mice burdening SK-Hep1 xenografts from HASP, MASP, LASP and PBS groups; **(B)** Representative images of SK-Hep1 subcutaneous xenograft tumors from HASP, MASP, LASP and PBS groups; **(C)** Comparison of average tumor volumes with time from HASP, MASP, LASP and PBS groups;** (D)** Comparison of average tumor weight from HASP, MASP, LASP and PBS groups; **(E)** Comparison of average weight in nude mice with time from HASP, MASP, LASP and PBS groups. Data were represented using the GraphPad Prism 5.02 software. *, *p* < 0.05, compared with the control group.

**Figure 5:**
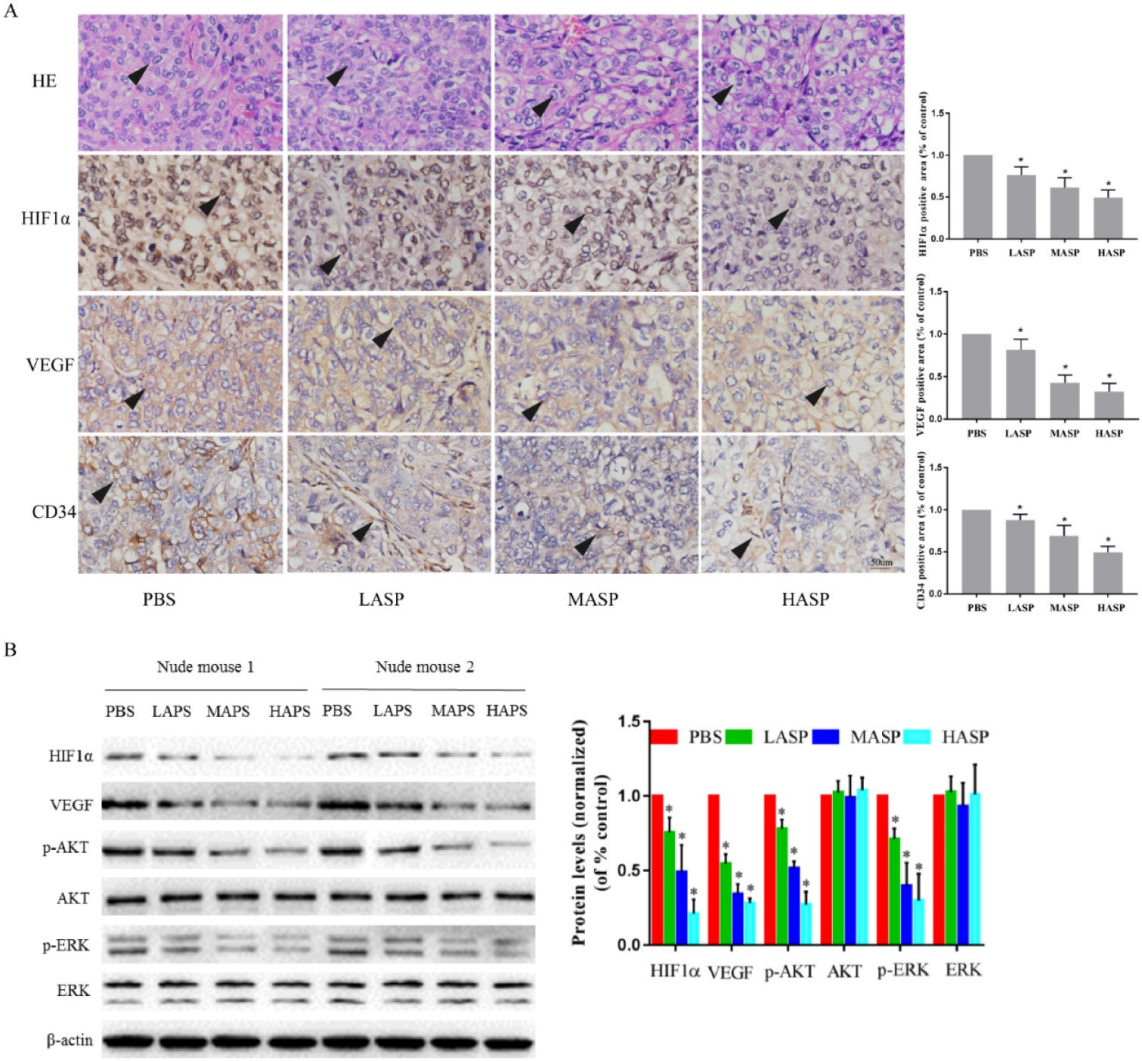
ASP promotes tumour cell apoptosis and suppresses angiogenesis of subcutaneous xenograft tumor tissues through regulating HIF1α/VEGF expression via MAPK and PI3K signaling pathway from nude mice. **(A)** H&E staining assayed effect of ASP on tumour cell apoptosis from subcutaneous xenograft tumor tissues using nude mice. IHC assayed effects of ASP on angiogenesis-related proteins, such as HIF-1α, VEGF and CD34, from subcutaneous xenograft tumor tissues using nude mice. Histogram presented the positive area per field. Scale bar, 100μm in Figure A. Black arrows indicating apoptotic tumour cells and protein expression of HIF-1α, VEGF and CD34. **(B)** Western blotting analysis assayed inhibitory effects of ASP on HIF-1α, VEGF, p-AKT, AKT, p-ERK and ERK proteins from subcutaneous xenograft tumor tissues using nude mice; Relative expression levels of HIF-1α, VEGF, p-AKT, AKT, p-ERK and ERK proteins from subcutaneous xenograft tumor tissues using nude mice were quantified using the ImageJ software. Data were graphed using GraphPad Prism. *, *p* < 0.05, compared with the control group.

In order to illustrate the effects of ASP on the growth and angiogenesis of tumors, we performed *in vivo* subcutaneous xenograft tumor experiment using nude mice. Our results showed that ASP regimes (HASP, MASP and LASP) significantly suppressed the growth of xenograft tumors on day 33 compared with the control group (PBS group) (Figure 4A-C). Additionally, ASP regimes (HASP and MASP) significantly decreased the weight of xenograft tumors compared with the control group (PBS group) (Figure 4D). We also found that ASP regimes (HASP, MASP and LASP) significantly prevented the tumor-mediated body weight loss in nude mice on day 30 and day 33 (Figure 4E). H&E staining showed that more apoptotic cells were found in xenograft tumor tissues of the ASP groups (HASP, MASP and LASP) compared with the PBS group (Figure 5A). The IHC results demonstrated that the staining of HIF-1α, VEGF and CD34 proteins in xenograft tumor tissues from nude mice administrated with ASP (HASP, MASP and LASP) was significantly weaker compared with the PBS group (Figure 5A). In addition, Western blotting analysis further indicated that ASP regimes (HASP, MASP and LASP) significantly suppressed the protein levels of HIF-1α, VEGF, p-AKT and p-ERK, while no significant changes were found in the protein levels of AKT and ERK (Figure 5B).

## Discussion

HCC is a type of common malignant tumor with high morbidity and mortality rate 1. Although many treatment strategies, including surgery, radiotherapy, chemotherapy, immunotherapy, molecular targeted therapy and TACE, have been applied for HCC patients, the OS of HCC patients still remains very poor 2.

Hypoxia is a common event in tumor tissues due to rapid growth of tumor cells, leading to insufficient blood supply. Hypoxia also promotes the over-expression of HIF-1α protein and its downstream VEGF protein in tumor cells. HIF-1α plays a pivotal role in promoting radiotherapy- and chemotherapy-resistance of malignant tumor cells 23-30, tumor angiogenesis 31-32, cancer cell metabolism 33-35, cell proliferation 36, 37, and cell migration and invasion 38-40. Our previous studies have also demonstrated that HIF-1α over-expression leads to unfavorable OS and promotes the proliferation, migration, invasion and angiogenesis of HCC cells 41. Excessive HIF-1α, which is produced due to hypoxia of tumor cells, might greatly contribute to the difficulty of tumor therapy. Therefore, suppression of HIF-1α in tumor cells presents a promising treatment strategy.

Epithelial-mesenchymal transition (EMT) play a pivotal part in migration and invasion of cancer cells 42, 43. Many studies have demonstrated that hypoxia could induce EMT of cancer cells by HIF-1α and subsequently enhance ability of migration and invasion 44, 45. Our previous studies have showed ASP could significantly inhibit migration of human HCC cells under hypoxia, and its mechanism might be achieved by reversing hypoxia-induced EMT 46. Our previous researches have also indicated ASP can suppress proliferation, migration, invasion and angiogenesis of HCC cells under normoxic conditions 20. Different from normoxia, hypoxia is more prone to lead to antitumor drug resistance and immune escape of tumor cells 13. So we need to furtherly confirm effects of ASP on the hypoxia-induced migration, invasion and angiogenesis of HCC cells and HIF-1α/VEGF signaling pathway, we performed this study.

Our results showed that ASP significantly inhibited hypoxia-induced proliferation, migration, invasion and angiogenesis of HCC cells. Mechanism study indicated that ASP significantly suppressed the expressions of HIF-1α and its downstream VEGF at the protein level partly via blocking MAPK and PI3K signaling pathway-related protein under hypoxic conditions. Moreover, subcutaneous xenograft tumor experiment in nude mice further demonstrated that ASP inhibited the growth and angiogenesis of subcutaneous xenograft tumor, and prevented the tumor-mediated weight loss of nude mice. Based on the inhibitory effect of ASP on HIF-1α protein under hypoxic conditions in HCC cells, we speculated that ASP might reverse radiochemotherapy resistance, enhance radiochemotherapy sensitivity, and suppress metastasis in HCC cells. We also planned to use a combination of ASP and oxaliplatin to improve the chemotherapy sensitivity in HCC cells and clarify its mechanism of action in subsequent study. Our final aim was to confirm that whether ASP, as an assisted anti-tumor drug, could improve OS and recurrence-free survival in HCC patients in future clinical trials.

Our previous studies showed ASP can suppress proliferation, migration, invasion and angiogenesis of HCC cells under normoxic conditions 20. Different from previous studies, this research indicated ASP can inhibit hypoxia-induced proliferation, migration, invasion and angiogenesis of HCC cells.

Our study still has some limitations. First, our results need to be verified by more HCC cell lines, such as the Huh7, bel7404 and SMMC-7721 cells. Second, the inhibitory effect of ASP on HIF-1α protein under hypoxic conditions needs to be confirmed using inhibitors of such signaling pathways. Moreover, we still need to further verify the anti-HCC effect of ASP in future clinical trials.

Collectively, our study demonstrated that ASP suppressed hypoxia-induced migration, invasion and angiogenesis of HCC cells partly through the down-regulation of HIF-1α/VEGF expression via the PI3K and MAPK signaling pathways. ASP, an extract of a widely used TCM with potent antitumor activity, deserved further research to determine its possible applications in the therapy of liver cancer.
